# Regulation of *Staphylococcus aureus* MntC Expression and Its Role in Response to Oxidative Stress

**DOI:** 10.1371/journal.pone.0077874

**Published:** 2013-10-28

**Authors:** Luke D. Handke, Julio C. Hawkins, Alita A. Miller, Kathrin U. Jansen, Annaliesa S. Anderson

**Affiliations:** Pfizer Vaccine Research, Pearl River, New York, United States of America; The Scripps Research Institute and Sorrento Therapeutics, Inc., United States of America

## Abstract

*Staphylococcus aureus* is a successful human pathogen that has developed several approaches to evade the immune system, including resistance strategies to prevent oxidative killing by immune cells. One mechanism by which this evasion occurs is by production of superoxide dismutase enzymes, which require manganese as a cofactor. Manganese is acquired by the manganese transporter MntABC. One component of this operon, MntC, has been proposed as a potential vaccine candidate due to its early *in vivo* expression and its ability to provide protection in preclinical models of staphylococcal infection. In the current study, we interrogate the role of this protein in protecting *S. aureus* from oxidative stress. We demonstrate that mutation of *mntC* in a number of invasive *S. aureus* clinical isolates results in increased sensitivity to oxidative stress. In addition, we show that while downregulation of *mntC* transcription is triggered upon exposure to physiological concentrations of manganese, MntC protein is still present on the bacterial surface at these same concentrations. Taken together, these results provide insight into the role of this antigen for the pathogen.

## Introduction

*Staphylococcus aureus* is the causative agent of diseases ranging from benign skin infections to life-threatening endocarditis, osteomyelitis, toxinoses, and bacteremia. Proliferation and virulence of this pathogen is dependent on its ability to scavenge essential metals which are normally sequestered by the host [1]. Historically, emphasis has been placed on the role of iron in *S. aureus* virulence (for recent reviews see [2]–[5]). However, recent reports suggest that manganese is also necessary for *S. aureus* virulence [6]–[8]. Resistance to oxidative stress by *S. aureus* can be attributed to the activity of superoxide dismutase enzymes, which use manganese as a cofactor and inactivate reactive oxygen species [9]–[11]. Corbin et al. demonstrated that calprotectin, a neutrophil-associated protein which chelates Mn^2+^, limits growth of *S. aureus* both *in vitro* and in abscesses [6]. Manganese chelation by calprotectin lowers *S. aureus* superoxide dismutase activity and renders the bacteria more sensitive to oxidative stress and killing by neutrophils [12].

ATP-binding cassette (ABC) transporters have been shown to play a central role in manganese transport in several species of bacteria [13]–[15]. However, the mechanism of manganese uptake by *S. aureus* is incompletely defined. *S. aureus* encodes an ABC transporter, MntABC, where *mntA* encodes the ATP-binding protein, *mntB* encodes the integral membrane protein, and *mntC* encodes the cation-binding lipoprotein. While *S. aureus* also encodes an additional manganese transporter, the Nramp homolog *mntH*, it appears that *S. aureus* imports manganese primarily by its ABC transporter [7]. Horsburgh et al showed that mutation of *mntA* in a laboratory isolate, 8325-4, resulted in a reduced growth rate and greater sensitivity to oxidative stress *in vitro* (although this strain background encodes a truncated copy of *mntH* [16]). In addition, it was shown that transcription of *mntABC* is down regulated by its cognate repressor, MntR, which, upon binding to Mn^2+^, blocks transcription of the *mntABC* operon. Deletion of *mntA* also resulted in slightly lowered virulence in a murine abscess model, but only when *mntH* was also insertionally inactivated [7].

MntC is a component of a tetravalent *S. aureus* vaccine currently under investigation in human clinical trials (U. S. National Institutes of Health Clinical Trial registry numbers NCT01364571 and NCT01643941) which, in addition to MntC, includes antigens designed to address immune evasion (capsular polysaccharide) and adhesion to host tissues (clumping factor A) [17]. It has recently been shown that MntC is a highly conserved protein among staphylococci; MntC homologs from 11 *Staphylococcus* species were found to share an average pairwise identity of 72% [18]. In addition, MntC was found to be surface expressed rapidly following infection of mice with a diverse group of *S. aureus* isolates [18]. Active vaccination with purified recombinant MntC significantly reduced both *S. aureus* and *S. epidermidis* bacterial burden in a mouse bacteremia model, suggesting that it may be an effective vaccine antigen for the prevention of *S. aureus* infection [18].

Here we present a brief report describing the precise regulation of *mntABC* expression by MntR and corresponding MntC protein levels. We also demonstrate that MntC is surface expressed at physiological concentrations of manganese and that it plays a key role in providing resistance to oxidative stress across diverse disease-causing clinical isolates.

## Materials and Methods

### Primers, strains, plasmids, and growth media

Primers, strains, and plasmids used in the current study are listed in Table 1. To make TSB-c medium, TSB (BD, Franklin Lakes, NJ) was stirred in the presence of 1% (w/v) Chelex 100 beads (Sigma-Aldrich, St. Louis, MO) for 4 hours. The medium was then filter-sterilized and supplemented with MgCl_2_ and Fe_(II)_SO_4_ to final concentrations of 50 µM and 1 µM, respectively. Inductively-coupled mass spectrometry analysis (performed by Robertson Microlit Laboratories, Ledgewood, NJ) of four independently prepared batches of the TSB medium that had been treated with Chelex 100 beads revealed that the medium contained 50 nM manganese ±17 nM.

**Table 1:** Primers, strains, and plasmids used in the study.

Primers:		
Primer name:	Sequence^a^:	
oLH431	GCT GCT GAA TTC TAT TCT AAA TGC ATA ATA AAT ACT G	
oLH432	TGT CAC TTT GCT TGA TAT ATG AG	
oLH398	CTC ATA TAT CAA GCA AAG TGA CAT TTA CTT GAC ATG ATA AAT ATT CTC	
oLH433	GTC ATA GAG AGT CCT CCT CTG AAT TAA AAG TTT AGG CTA ACC TAA TTA ATT G	
oLH434	CAG AGG AGG ACT CTC TAT GAC	
oLH435	GCT GCT GGA TCC TCA ATG TAT CTT ATC ATG TCT GC	
oLH442	GTC ATA GAG AGT CCT CCT CTG TGT CAC TTT GCT TGA TAT ATG AG	
oLH443	CTC ATA TAT CAA GCA AAG TGA CAC AGA GGA GGA CTC TCT ATG AC	
oLH202	GCT GCT GGA TCC AAG AAA GCG GTA AAA CTA ATT CC	
oLH203	GTA GCA TGT CTC ATT CAA TTT AAG GAT TGC CTT TAA ATA GTC C	
oLH204	GGA CTA TTT AAA GGC AAT CCT TAA ATT GAA TGA GAC ATG CTA C	
oLH205	GCA AAC ATG TTC ATT GCA TTT TCA AAA CTG GTT TAA GCC GAC	
oLH206	GTC GGC TTA AAC CAG TTT TGA AAA TGC AAT GAA CAT GTT TGC	
oLH207	GCT GCT GTC GAC AAT TCA CTA TTT CAG GGT TGG C	
oLH81	GCT GCT GGA TCC ACA CTA CGA CAG ATT TGT ACC	
oLH82	CCG TTC CCA ATT CCA CAT TGT TAC CAG CCT GTT CTA AGG C	
oLH83	GCC TTA GAA CAG GCT GGT AAC AAT GTG GAA TTG GGA ACG G	
oLH84	GGT TTA ACA TCT TTT GAT ACT GCT TAA CAA TTA TTA GAG GTC ATC G	
oLH85	CGA TGA CCT CTA ATA ATT GTT AAG CAG TAT CAA AAG ATG TTA AAC C	
oLH86	GCT GCT GGA TCC AAC TTC TAG CTT TTC TCT TTC G	
Strains:		
Strain name:	Relevant characteristics:	Source or reference:
MRSA252	Template for pI258 transcriptional terminator (TT)	[37]
Newman	Template for P*_mntABC_* promoter fragment	[38]
COL	Template for mntR-1, mntR-2, mntC-1, and mntC-2 fragment for gene knockout constructs	[39]
RN4220	Restriction-deficient RN450	[40]
RN4220 *mntC*::*neo*	*mntC* knockout strain of RN4220	This study
RN4220 *mntR*::*ermC*	*mntR* knockout strain of RN4220	This study
RN4220 *geh*::pLH75	pLH75 integrated at the *geh* locus by L54a integrase encoded on pLH69, which was subsequently cured from the strain	This study
RN4220 *geh*::pLH76	pLH76 integrated at the *geh* locus by L54a integrase encoded on pLH69, which was subsequently cured from the strain	This study
8325-4	NCTC8325 cured of three phophage	[41]
8325-4 *geh*::pLH75	*geh*::pLH75 allele from RN4220 *geh*::pLH75 transduced into 8325-4	This study
8325-4 *geh*::pLH76	*geh*::pLH76 allele from RN4220 *geh*::pLH76 transduced into 8325-4	This study
8325-4 *mntC*::*neo*	*mntC*::*neo* allele moved from RN4220 *mntC*::*neo* by transduction	This study
8325-4 *mntR*::*ermC*	*mntR*::*ermC* allele moved from RN4220 *mntR*::*ermC* by transduction	This study
8325-4 *mntR*::*ermC geh*::pLH76	*mntR*::*ermC* allele from RN4220 *mntR*::*ermC* introduced into 8325-4 *geh*::pLH76 by transduction	This study
PFESA0179	*S. aureus* clinical isolate, CC5	This study
PFESA0190	*S. aureus* clinical isolate, CC5	This study
PFESA0237	*S. aureus* clinical isolate, CC8	This study
PFESA0105	*S. aureus* clinical isolate, CC22	This study
PFESA0164	*S. aureus* clinical isolate, CC30	This study
PFESA0186	*S. aureus* clinical isolate, CC30	This study
PFESA0005	*S. aureus* clinical isolate, CC45	This study
PFESA0113	*S. aureus* clinical isolate, CC45	This study
PFESA0176	*S. aureus* clinical isolate, CC45	This study
PFESA0106	*S. aureus* clinical isolate, CC45	This study
PFESA0175	*S. aureus* clinical isolate, CC45	This study
PFESA0119	*S. aureus* clinical isolate, CC8, USA300	This study
Plasmids:		
Plasmid name:	Relevant characteristics:	Source:
pRL-null	Template for *Renilla* luciferase, *Rluc*	Promega
pLH69	pNL9164 with the L54a *int* gene cloned in at the *Sph*I and *Kpn*I sites	This study
pLH71	*attP-tet* cassette cloned into the *Aat*II site in pUC19	This study
pLH75	TT-R*luc* cassette cloned into the integrative vector, pLH71, at the *Eco*RI/*Bam*HI sites.	This study
pLH76	TT-P*_mntABC_*-R*luc* cassette cloned into the integrative vector, pLH71, at the *Eco*RI/*Bam*HI sites.	This study
pLH26	*mntC*::*neo* cassette cloned into pSPT181 at the *Bam*HI site.	This study
pLH47	*mntR*::*ermC* cassette cloned into pSPT181 at the *Bam*HI/*Sal*I sites.	This study
pE194	Template for *ermC*	[42]
pSPT181	Temperature-sensitive *E. coli*-*S. aureus* shuttle vector	[43]
pUB110	Template for *neo*	ATCC

### Construction of a mntC knockout strain

A *mntC*::*neo* knockout cassette consisting of two 800 bp fragments (mntC-1 and mntC-2) flanking the neomycin resistance gene, *neo*, was constructed. The primer sets and DNA templates used to amplify each of the fragments were: (i) mntC-1: oLH81/oLH82, *S. aureus* COL genomic DNA; (ii) *neo*: oLH83/oLH84, pUB110; (iii) mntC-2: oLH85/oLH86, *S. aureus* COL genomic DNA. All PCRs were performed with iProof polymerase (Bio-Rad, Hercules, CA), and all cloning and molecular biology techniques were performed as described [19]. After purification, these three PCR products were spliced together by amplification with oLH81 and oLH86, and the knockout cassette was cloned into pSPT181 at the *Bam*HI site. After electroporation and chromosomal integration into *S. aureus* RN4220, the final *mntC*::*neo* mutant strain was obtained after plasmid excision by secondary recombination. The mutation was introduced into other *S. aureus* isolates by transduction using protocols described previously [20]. In the resulting neomycin resistant transductants, insertional inactivation of *mntC* was confirmed by PCR. Mutants were shown to be of the expected strain lineage with a Qualicon RiboPrinter (DuPont, Wilmington, DE).

### Construction of a mntR knockout strain

A *mntR*::*ermC* knockout cassette, consisting of two ∼1.2 kb fragments (mntR-1 and mntR-2) flanking the erythromycin resistance gene, *ermC*, was constructed. The primer sets and DNA templates used to amplify each of the fragments were: (i) mntR-1: oLH202/oLH203, *S. aureus* COL genomic DNA; (ii) *ermC*: oLH204/oLH205, pE194; (iii) mntR-2: oLH206/oLH207, *S. aureus* COL genomic DNA. After purification, these three products were spliced together by amplification with oLH202 and oLH207, and the knockout cassette was cloned into pSPT181 at the *Bam*HI and *Sal*I sites. After electroporation and chromosomal integration into *S. aureus* RN4220, the final *mntR*::*ermC* mutant strain was obtained after plasmid excision by secondary recombination. This mutation was transduced into *S. aureus* 8325-4 *geh*::pLH76 essentially as described elsewhere [20]. In the resulting erythromycin resistant transductants, insertional inactivation of *mntR* was confirmed by PCR. Mutants were shown to be of the expected strain lineage with a Qualicon RiboPrinter.

### Construction of TT-P_mntABC_-Rluc and TT-Rluc reporter fusions and reporter assays

To more easily monitor the levels of *mntABC* promoter activity, a transcriptional fusion was constructed comprised of the *Renilla* luciferase (*Rluc*) gene placed under the control of the *mntABC* promoter (P*_mntABC_*). To prevent non-specific read-through transcription, a transcriptional terminator (TT) from pI258 (as described in [21]) was amplified with primers oLH431 and oLH432. The P*_mntABC_* promoter fragment, which included the MntR binding box sequences [7], [22], was amplified from *S. aureus* Newman genomic DNA with primers oLH398 and oLH433. The promoterless *Rluc* reporter gene was amplified from pRL-null (Promega, Madison, WI) with primers oLH434 and oLH435. Each of these products was purified (QIAquick PCR Purification Kit, Qiagen, Valencia, CA) and were spliced together by amplification with primers oLH431 and oLH435 to give TT-P*_mntABC_*-*Rluc*. A promoterless version of this cassette, TT-*Rluc*, was constructed with PCR primers oLH431 and oLH442 for TT and oLH443 and oLH435 for *Rluc*. These two PCR products were purified and spliced together as before to yield TT-*Rluc*. The TT-*Rluc* and TT-P*_mntABC_*-*Rluc* cassettes were cloned into the L54a *attP*-encoding integrative vector, pLH71 (see Table 1 for more detail), at *Eco*RI and *Bam*HI sites yielding pLH75 and pLH76, respectively. These plasmids were transformed into *S. aureus* RN4220/pLH69 by electroporation, where pLH69 encodes the phage L54a integrase [23]–[26]. After integration of the plasmids into the chromosome at *geh* was confirmed, pLH69 was cured from the strain, and the cassettes were transduced into *S. aureus* strains 8325-4 and 8325-4 *mntR*::*ermC* essentially as described elsewhere [20].

*S. aureus* strains with chromosomally integrated reporters bearing either a promoterless *Rluc* (8325-4 *geh*::pLH75) or P*_mntABC_*-*Rluc* (8325-4 *geh*::pLH76) were cultured at 37°C in TSB-c medium to mid-exponential phase of growth, then subcultured into the same medium containing increasing concentrations of supplemental MnSO_4_ to a final optical density at 600 nm (OD_600_) of 0.01. After one hour incubation with shaking at 37°C, 40 µL of each culture was mixed with 10 µL of 5X *Renilla* luciferase lysis buffer (Promega) in an opaque white 96-well plate, and the plate was shaken for two minutes at 200 rpm at room temperature. Fifty µL of *Renilla* luciferase substrate (Promega) was added and the samples were shaken for an additional two minutes before measuring relative luminescence on a Spectramax M5e (Molecular Devices, Sunnyvale, CA).

### Western blot hybridization for MntC

Cells were grown overnight in TSB-c at 37°C with shaking, and subcultured (1:200 dilution) the following day in fresh TSB-c supplemented with the indicated concentration of MnSO_4_. Cells were harvested in the mid-exponential phase of growth, lysed with lysostaphin, and proteins were denatured in SDS-PAGE loading buffer. Following electrophoretic fractionation and transfer, Western blots were probed with murine anti-MntC 78-7 monoclonal antibody (at 3 µg/mL) as the primary antibody, and goat anti-mouse IgG (H+L)-AP Conjugate (Bio-Rad; diluted 1:5,000) as the secondary antibody. Western blots were processed using standard protocols [27].

### Assessment of MntC surface expression by whole cell ELISA

Selected clinical isolates from each clonal complex and their cognate *mntC*::*neo* mutant derivatives were grown in 5 mL TSB-c overnight at 37°C with shaking. The next day, cultures were diluted 1∶200 into TSB-c containing 400 nM MnSO_4_. Cultures were shaken at 37°C until they reached an OD_600_ = 0.6 – 0.9. An OD_600_ equivalent of 5 was collected, pelleted by centrifugation, and the supernatant was decanted. Cells were resuspended in 1 mL of 1X DPBS (Invitrogen, Carlsbad, CA) +20% glycerol and frozen at −70°C until use. Frozen cells were diluted 1∶10 in 1X DPBS, and 100 µL of each strain of *S. aureus* was added per well to a NUNC Maxisorp plate (Thermo Fisher Scientific, Waltham, MA). Plates were incubated at 4°C for 16 hours. The plate was blocked with 25 µL of porcine serum (J R Scientific, Woodland, CA) per well, and plates were incubated at 4°C for an additional 2 hours. Plates were washed with 300 µL/well three times with TBS containing 0.1% Brij-35 (CellGro/Mediatech, Manassas, VA). One hundred µL of 4 mg/mL IgG-purified polyclonal antibodies from either naïve or anti-MntC rabbit sera [18] diluted 1∶800 in 20% porcine serum in sterile 1X DPBS was added to each well and incubated at 4°C for 2 hours. Plates were washed three times as described above, then 100 µL of goat anti-rabbit-AP secondary antibody (Southern Biotech, Birmingham, AL) diluted 1∶1000 in 20% porcine serum was added to each well and plates were incubated at room temperature for 1 hr. Plates were then washed three times as described above, followed by the addition of 100 µL Alkaline Phosphatase Reagent for ELISA (Sigma-Aldrich). Plates were incubated for 15 min at room temperature and optical densities at 405 nm were determined. The experiment was performed four times, and each of these experiments was done with an independently grown panel of *S. aureus* cells.

### Assessment of MICs to methyl viologen

The Minimal Inhibitory Concentration (MIC) of methyl viologen (Sigma-Aldrich) necessary to inhibit bacterial growth was determined by broth microdilution according to standard protocols [28] with either TSB-c or TSB-c supplemented with 10 µM MnSO_4_ as the growth medium. MIC determinations were made during at least two independent assays.

## Results and Discussion

### *mntABC* transcription and MntC expression are observed at physiological levels of Mn^2+^

The US Department of Health reports that the normal range for manganese levels in humans is ∼4 to 15 µg/L in blood, which is equivalent to 73 nM to 273 nM [29]. It has been reported that manganese-mediated repression of *mntABC* transcription occurs via MntR [7]. However, the manganese concentrations tested were higher than levels normally encountered in human blood (≥1 µM) and therefore higher than what *S. aureus* would normally encounter during bacteremia. We therefore sought to more clearly define the concentrations of Mn^2+^ that regulate *mntABC* at the level of transcript and protein expression. A transcriptional fusion of the *mntABC* promoter with *Rluc* was constructed to facilitate precise definition of the manganese concentration required for the regulation of *mntABC* transcription.

**Figure 1:**
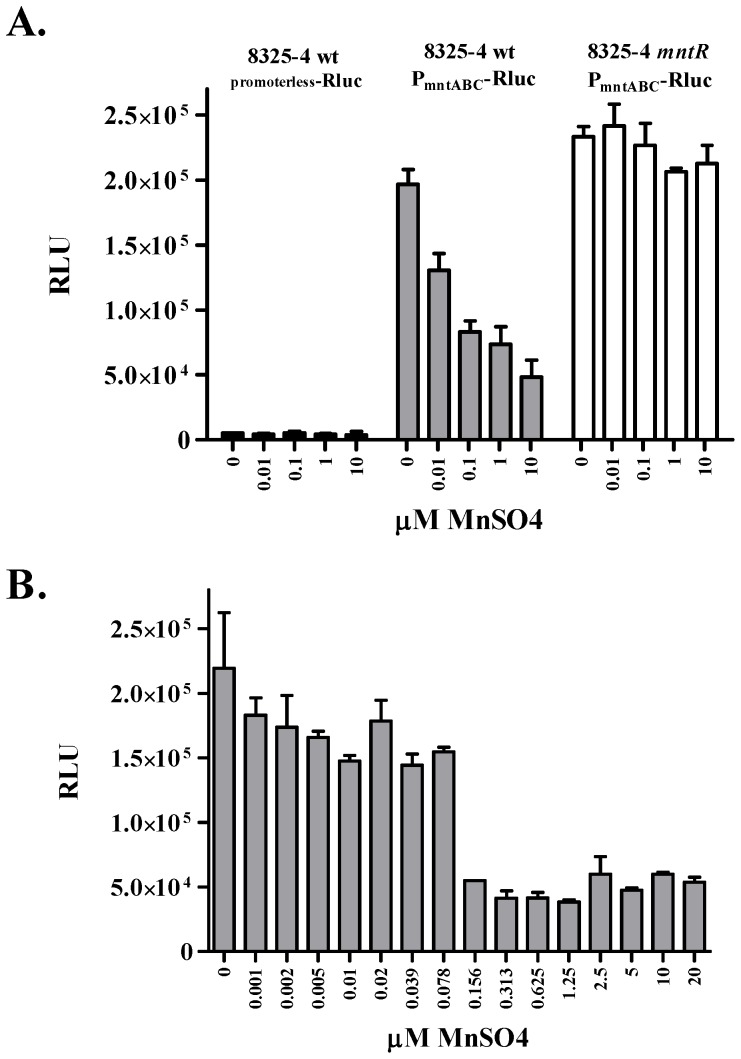
Promoter activity of *S. aureus* P*_mntABC_* in response to Mn^2+^. Panel A: Luciferase reporter activity of *S. aureus* 8325-4 *geh*::pLH75 (*Rluc* without a promoter, black bars), *S. aureus* 8325-4 *geh*::pLH76 (P*_mntABC_*-*Rluc*, gray bars), and *S. aureus* 8325-4 *mntR*::*ermC geh*::pLH76, (P*_mntABC_*-*Rluc* with *mntR* deleted, white bars) in the presence of increasing Mn^2+^ as revealed by luminescence from the *Rluc* reporter. Panel B: *mntABC* promoter activity of 8325-4 *geh*::pLH76 in the presence of two-fold serial dilutions of Mn^2+^ as revealed by luminescence from the *Rluc* reporter. Error bars indicate one standard deviation from the mean of triplicate wells.

*S. aureus* strains with chromosomally integrated reporters bearing either a promoterless *Rluc* (*S. aureus* 8325-4 *geh*::pLH75) or P*_mntABC_*-*Rluc* (8325-4 *geh*::pLH76) were cultured in TSB-c medium containing various concentrations of supplemental MnSO_4_. The promoterless *Rluc* strain had minimal levels of reporter activity at all concentrations of MnSO_4_ tested (Figure 1A). In contrast, *S. aureus* 8325-4/ P*_mntABC_*-*Rluc*, which contained *Rluc* under the control of a 67 bp *mntABC* promoter fragment, exhibited high levels of reporter activity in the absence of supplemental MnSO_4_. Consistent with previously published results [7], reporter activity decreased as a function of increasing MnSO_4_ in the culture.

The observed repression of reporter activity in the presence of manganese is likely due to the metalloregulatory protein, MntR, responding to increasing Mn^2+^ as described previously [7]. To confirm this hypothesis, a *mntR* knockout mutant was constructed. As shown in Figure 1A, deletion of *mntR* relieved the manganese-dependent repression of the *P_mntABC_-Rluc* reporter. These results indicate that expression and regulation of this promoter-reporter system work in a similar fashion to what has been published previously [7]. Unlike the earlier study that utilized a *mntA-lacZ* fusion containing a 578 bp promoter fragment, these results illustrate that a 67 bp P*_mntABC_* fragment is sufficient to support both expression of *mntABC* and Mn^2+^-dependent regulation by MntR.

To determine the minimum concentration of manganese at which expression of *mntABC* was repressed by MntR, *S. aureus* 8325-4/ P*_mntABC_*-*Rluc* was cultured in the presence of two-fold serial dilutions of Mn^2+^ (Figure 1B). At concentrations of 78 nM MnSO_4_ and below, levels of reporter activity were high (between 1.5×10^5^ and 2×10^5^ relative light units, RLU). Higher concentrations triggered repression of reporter activity, and maximal repression was observed at concentrations of ≥156 nM MnSO_4_. Despite this repression, reporter activity was still observed, indicating that, under these growth conditions, MntR only down regulates expression without abolishing it. These results indicate that *S. aureus* responds to extracellular concentrations of manganese in the range found in human blood (73 nM to 273 nM). The observation that *mntABC* transcription is not completely repressed at these concentrations correlates well with *in vivo* expression of MntC [18]. The Mn^2+^ concentration at which promoter repression occurs also correlates with the experimentally determined binding affinity of purified MntC for Mn^2+^, which is in the nanomolar range [30].

**Figure 2:**
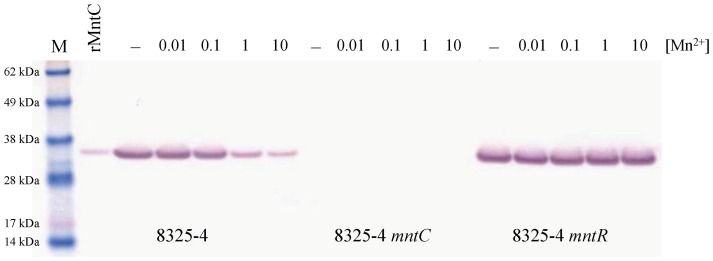
Western blot expression of MntC in *S. aureus* cultures supplemented with manganese. *S. aureus* strains 8325-4, 8325-4 *mntC*, and 8325-4 *mntR* were grown in TSB-c medium in the presence of increasing concentrations of manganese. Cells were grown in the absence of supplemental manganese (minus sign) or in the presence of 0.01 µM, 0.1 µM, 1 µM, or 10 µM manganese as indicated, and samples of crude lysates were resolved by SDS-PAGE. Recombinant expressed MntC (rMntC) and protein molecular weight standards (M) are included for reference.

To determine the degree to which manganese-dependent repression of *mntABC* RNA expression impacts MntC protein production, *S. aureus* 8325-4 wild-type, 8325-4 *mntC*, and 8325-4 *mntR* strains were cultured in TSB-c supplemented with increasing concentrations of Mn^2+^. As shown in Figure 2, expression of MntC protein was highest in the absence of manganese. In addition, there was a clear down-regulation of MntC protein levels in the *S. aureus* 8325-4 wild-type strain as a function of increasing levels of Mn^2+^ in the culture medium. As expected, no MntC protein was detectable in *S. aureus* 8325-4 *mntC* at any concentration of Mn^2+^ tested. For *S. aureus* 8325-4 *mntR*, levels of MntC protein were high and not affected by the concentration of manganese in the growth medium. These results indicate that, although manganese-mediated repression of *mntABC* transcription by MntR lowers the concentration of MntC protein, it is still expressed at the manganese concentrations which were tested under these growth conditions.

### MntC is present on the surface of diverse *S. aureus* clinical isolates

**Figure 3:**
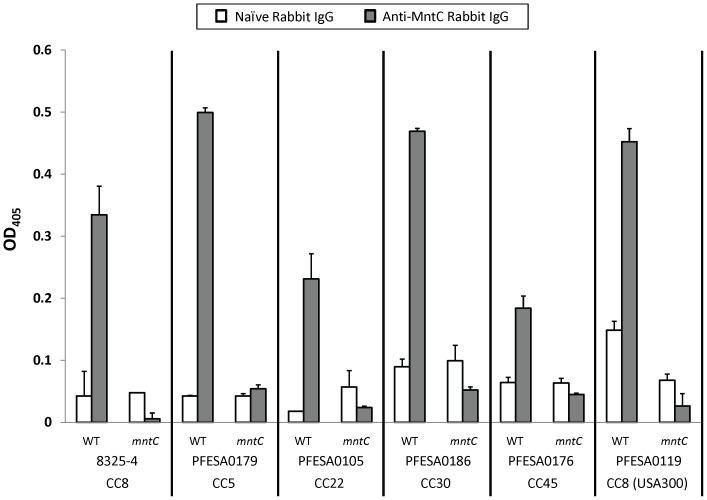
Relative surface expression of MntC across *S. aureus* clinical isolates as measured by whole cell ELISA. *S. aureus* clinical isolates (WT) representing five different clonal complexes and their isogenic *mntC* mutants (*mntC*) were grown in TSB-c supplemented with 400 nM MnSO_4_, then tested by whole cell ELISA for relative expression of MntC using polyclonal antibodies purified either from naïve rabbits (unshaded bars) or rabbits immunized with purified MntC (shaded bars). Wild-type 8325-4 and its isogenic *mntC* mutant were evaluated as comparators. Results were plotted following background correction: average background signal (from assays performed without primary naïve rabbit IgG or primary anti-MntC antibodies) were subtracted from the average sample signals from assays performed with primary antibody. Error bars indicate the standard deviation from duplicate samples. Representative data from one of four experiments yielding similar results is shown (see Materials and Methods).

Although *mntC* has been shown to be highly conserved across *S. aureus* isolates, its role in bacterial fitness, virulence and resistance to neutrophil killing in clinically relevant disease-causing isolates has yet to be demonstrated. In order to show that, (1) the results described above with the laboratory strain 8325-4 could be extended to clinically relevant strains, and (2) the levels of MntC detected by Western Blot correlate with surface expression, we performed whole cell ELISAs using anti-MntC polyclonal antibodies on representative clinical isolates grown *in vitro*. The isolates were selected from the five *S. aureus* clonal complexes most often associated with global disease: CC5, CC8, CC22, CC30, and CC45 [31]. As shown in Figure 3, we found that MntC was detected on the surface of each of the clinical isolates tested and that the level of expression for three of the five strains was equivalent to that observed in the 8325-4 laboratory strain.

### Inactivation of *mntC* results in sensitivity to oxidative stress across *S. aureus* clinical isolates

Polymorphonuclear neutrophils are important cellular mediators of the immune response to *S. aureus*. These immune cells are adept at killing engulfed bacteria through the production of a respiratory burst, i.e., a potent cocktail of superoxide radicals, hydrogen peroxide, and hydroxyl radicals that is secreted into the phagosome [32]. Methyl viologen (or paraquat), a quaternary ammonium compound which generates intracellular superoxide [33], [34], is frequently used *in vitro* to mimic the oxidative stress that the bacteria encounter during the respiratory burst. Increased sensitivity to methyl viologen was observed with a *S. aureus mntA* mutant made in the laboratory strain background, 8325-4 [7]. This mutation in the first gene in the operon (*mntA*) likely resulted in polar effects that disrupt expression of the downstream genes *mntB* and *mntC*. In addition, it has been shown that *S. aureus* 8325-4 encodes a truncated copy of the Nramp homolog *mntH* [16], so it cannot be concluded that the mutation in *mntA* was the sole cause of the observed sensitivity to methyl viologen (i.e., the observed phenotype may require mutation of both manganese transport systems). As a result, experiments were performed to look specifically at the contribution of the MntC vaccine antigen to *S. aureus* sensitivity to oxidative stress. Further, given that the previous observation was made with only a single laboratory strain of *S. aureus*, it was important to determine whether *mntC*-inactivation was similarly important for resistance to oxidative stress in diverse *S. aureus* strains that cause human disease. To test this possibility, a *mntC* mutation was transduced from *S. aureus* RN4220 into twelve clinical isolates from nosocomial infections representing different clonal complexes, including the five most often associated with invasive disease. Among the 12 isogenic pairs of clinical isolates were two MRSA strains, including one USA300, a lineage associated mainly with community-acquired infections. In order to quantitate the effect of *mntC* inactivation, minimum inhibitory concentrations (MICs) of methyl viologen were determined for wild-type and *mntC* isogenic strains in Mn^2+^-depleted media (Table 2). Without exception, all *mntC* strains were more sensitive to methyl viologen than their wild-type counterparts. Relative to the respective wild type strains, the methyl viologen MICs were at least 8-fold lower in the *mntC* mutants. When *mntC* was supplied in *trans*, this effect was reversed and resistance to methyl viologen was restored to wild-type levels (data not shown). Further, resistance to methyl viologen was restored when the *mntC* mutant strains were cultured in the presence of manganese (Table 2). These data demonstrate that increased sensitivity to oxidative stress is a general characteristic when *mntC* is disrupted in *S. aureus*. We conclude that the MntC plays a central role in resistance to oxidative stress across invasive *S. aureus* clinical isolates.

**Table 2:** Sensitivity of genetically diverse *S. aureus mntC* strains to oxidative stress.

			MV MIC (mM)^a^ TSB-c			MV MIC (mM)^a^ TSB-c + Mn^b^	
Strain	Origin	Clonal Complex	*wt*	*mntC*	Fold Change in Sensitivity	*wt*	*mntC*
8325-4	Lab. isolate	CC8	25	0.2	125	25	25
PFESA0179	UK	CC5	25	1.56	16	25	25
PFESA0190	UK	CC5	25	1.56	16	25	25
PFESA0237	UK	CC8	50	6.25	8	25	25
PFESA0105	Netherlands	CC22	25	0.39	64	25	25
PFESA0164	UK	CC30	12.5	0.78	16	12.5	12.5
PFESA0186	UK	CC30	25	0.39	64	25	25
PFESA0005	USA	CC45	50	0.78	64	25	25
PFESA0113	UK	CC45	25	3.12	8	25	25
PFESA0176	UK	CC45	25	3.12	8	25	25
PFESA0106	Canada	CC45	12.5	0.78	16	12.5	12.5
PFESA0175	Finland (MRSA)	CC45	50	0.78	64	25	25
PFESA0119	USA (MRSA)	CC8 (USA300)	25	0.39	64	25	25

a MV MIC: minimum inhibitory concentration of methyl viologen.

b TSB-c + Mn: TSB-c supplemented with 10 µM MnSO_4_.

### MntC regulation and its role in resistance to oxidative stress

In the current study, we sought to further define the regulation of MntC and its role in resistance to oxidative stress in *S. aureus*. Using a chromosomally-integrated *mntABC* promoter-reporter fusion, we demonstrated that transcription of *mntABC* is repressed in the presence of Mn^2+^ in a MntR-dependent manner. Further, we showed that down-regulation of the promoter-reporter fusion occurred at physiologically relevant concentrations of manganese [29]. Under these growth conditions, expression from the *mntABC* promoter was down regulated at Mn^2+^ concentrations greater than 156 nM. However, complete repression was never observed, even at micromolar concentrations of manganese. In contrast, Horsburgh *et al*, reported complete repression of *mntABC* at very high concentrations of manganese by Northern blot analysis (≥10 µM) [7]. This discrepancy could be explained either by the relative sensitivity of the *Rluc* reporter construct as compared to a Northern blot, or by the potential contribution of additional negative regulators whose binding sites were not present in the 67 bp promoter fragment used in our construct. We also found that MntC was surface expressed at 400 nM MnSO_4_ in *S. aureus* clinical isolates representative of the most prevalent disease-causing strains. Finally, *mntC* mutation correlated with heightened sensitivity to oxidative stress in a wide spectrum of clinical isolates.

Several antigens have been proposed as potential vaccine components for the prevention of staphylococcal infections [35]. Because manganese plays an important role in *S. aureus* virulence, antibodies resulting from vaccination with MntC have the potential to prevent MntC-mediated uptake of Mn^2+^ by *S. aureus*, thereby rendering the organism more sensitive to oxidative killing by host immune cells. The results presented above, in combination with the high degree of protein sequence conservation and the demonstration of surface expression during murine models of infection [18] and human infection [36] make MntC an interesting candidate for assessment as a *S. aureus* vaccine antigen.
